# Agricultural diversification as an important strategy for achieving food security in Africa

**DOI:** 10.1111/gcb.14158

**Published:** 2018-04-25

**Authors:** Katharina Waha, Mark T. van Wijk, Steffen Fritz, Linda See, Philip K. Thornton, Jannike Wichern, Mario Herrero

**Affiliations:** ^1^ CSIRO Agriculture & Food St Lucia QLD Australia; ^2^ Livestock Systems and the Environment International Livestock Research Institute (ILRI) Nairobi Kenya; ^3^ International Institute for Applied Systems Analysis (IIASA) Laxenburg Austria; ^4^ CGIAR Research Program on Climate Change, Agriculture and Food Security (CCAFS), ILRI Nairobi Kenya; ^5^ Plant Production Systems Wageningen University & Research Wageningen the Netherlands

**Keywords:** coefficient of variation, crop production, farming diversity, food availability, livestock production

## Abstract

Farmers in Africa have long adapted to climatic and other risks by diversifying their farming activities. Using a multi‐scale approach, we explore the relationship between farming diversity and food security and the diversification potential of African agriculture and its limits on the household and continental scale. On the household scale, we use agricultural surveys from more than 28,000 households located in 18 African countries. In a next step, we use the relationship between rainfall, rainfall variability, and farming diversity to determine the available diversification options for farmers on the continental scale. On the household scale, we show that households with greater farming diversity are more successful in meeting their consumption needs, but only up to a certain level of diversity per ha cropland and more often if food can be purchased from off‐farm income or income from farm sales. More diverse farming systems can contribute to household food security; however, the relationship is influenced by other factors, for example, the market orientation of a household, livestock ownership, nonagricultural employment opportunities, and available land resources. On the continental scale, the greatest opportunities for diversification of food crops, cash crops, and livestock are located in areas with 500–1,000 mm annual rainfall and 17%–22% rainfall variability. Forty‐three percent of the African cropland lacks these opportunities at present which may hamper the ability of agricultural systems to respond to climate change. While sustainable intensification practices that increase yields have received most attention to date, our study suggests that a shift in the research and policy paradigm toward agricultural diversification options may be necessary.

## 1 INTRODUCTION

Achieving global food security remains a key challenge for the future, particularly given continued population increases, dietary shifts, and global climate change. Attention has been largely focused on agricultural intensification as a mechanism for producing more, even though food insecurity in many places is largely an income and distribution problem (Hazell & Wood, 2008). Also, there has been much less research focusing on the contribution of farming diversity toward achieving food security, despite evidence that more diverse agroecosystems are likely to perform better today and under changing environmental conditions because a broader range of functions and responses to change will stabilize the system (Altieri, 1999; Lin, 2011; Michler & Josephson, 2017).

Farmers in Africa have long adapted to climatic and other risks by diversifying their farming activities (Ebi et al., 2011; Smith, 1990), which may increase their ability to cope with change. This can happen by spreading the risk among different crop and livestock types (Antwi‐Agyei, Stringer, & Dougill, 2014; Below et al., 2012; Bryan et al., 2013; Mary & Majule, 2009; Waha et al., 2013), income diversification (Block & Webb, 2001) or by increasing the range of agricultural products for markets or subsistence (McCord, Cox, Schmitt‐Harsh, & Evans, 2015). Selling own products is also very important for overall food security outcomes for farmers in sub‐Saharan Africa. Purchased food accounts for a large proportion of household consumed calories, for example, in Ethiopia one third to more than half of all calories (Sibhatu & Qaim, 2017). Eighty‐three percentage of farm households in sub‐Saharan Africa sell part of their crop produce, sometimes even before they produce enough to be self‐sufficient (Frelat et al., 2016). Also many African farmers own livestock as an insurance during periods of drought (Kazianga & Udry, 2006). One way of measuring agricultural diversity is to assess the crop and farming diversity, that is, the number of crops grown and the number of overall farming activities including livestock husbandry.

The aim of this paper is to establish the relationship between diversity and food availability for Africa both at continental and household levels and to identify areas of low and high farming diversity using basic climatology. For this, we are using information from more than 28,000 agricultural household surveys and spatially explicit data on crop and livestock production. Crop and farming diversity are related to rainfall and rainfall variability (Bezabih & Sarr, 2012; Bhatta, Aggarwal, Shrivastava, & Sproule, 2016; Rufino et al., 2013) in that very low rainfall, very high rainfall variability, and high total rainfall will limit agriculture. Thus, by using information on rainfall, land cover and spatially explicit data on crop presence and livestock production, we map the spatial distribution of farming diversity in Africa to highlight areas with potentially limited options of switching to an alternative farming activity under adverse climatic conditions. Increased understanding of where diversification potentials are high and where farmers are more or less likely to adapt to a changing climate can inform risk management strategies as part of a climate‐related risk assessment for the African continent.

## 2 MATERIALS AND METHODS

### 2.1 Climate data

Rainfall data were obtained from the WorldClim version 1.4 (release 3) dataset (Hijmans, Cameron, Parra, Jones, & Jarvis, 2005), which contains monthly rainfall climatology from 1950 to 2000 for the entire globe at a 30 arc second resolution (~1 km). The data have been calculated as means of all rainfall observations from weather stations from various sources interpolated to climate surfaces. The rainfall data were aggregated to a 30 arc minute (~50 km) resolution. Rainfall variability, measured as the coefficient of variation (CV) of annual rainfall for the same period, was estimated using the weather generator MarkSim (Jones & Thornton, 1999, 2013). The coefficient of variation is a measure of relative variability, that is, the variation in rainfall does not depend on total rainfall and is useful because we are comparing locations in different climate zones.

The rainfall data were classified into 41 equal intervals ranging from 0 to 3,000 mm rainfall in 100 mm steps, where the leftmost interval corresponds to class one, the next leftmost to class two and so on, and the intervals are closed on the right and open on the left (e.g., class 700–800 mm does include values larger than 700 mm and equal to or lower than 800 mm). Similarly, rainfall variability data were classified into 32 equal intervals ranging from 10% to 90% CV of rainfall in 2.5% steps.

### 2.2 Land cover

Land cover from GLC2000 (Fritz et al., 2003) and MODIS (Friedl et al., 2010; MCD12Q1 Collection 5) land cover products were used and their land cover classes simplified as shown in Table S2. The MODIS land cover product was generated using an ensemble supervised classification algorithm and training data from 1,860 sites across the World's land areas. GLC2000 is a harmonized land cover product from 19 World regions based on imagery from the SPOT‐4 VEGETATION instrument. The GLC2000 was generated at a 1 km spatial resolution for the reference year 2000, while the MODIS land cover product for 2001 is available at a 500 m resolution. Both data sets were aggregated to a 30 arc minute resolution.

### 2.3 Crop area and livestock production data for continental analysis

Crop area for 23 crops and crop groups was obtained from M3‐Crop (Monfreda, Ramankutty, & Foley, 2008) for 1998–2002 and Map‐SPAM 2000 (version 3.0.6/2012; You et al., 2013) for 1999–2001 on a 5 arc minute resolution and aggregated to 30 arc minutes. M3‐Crop reports harvested area from 175 crops and 11 crop groups as fractions of the grid cell area. Map‐SPAM 2000 reports harvested and physical area from 20 crops.

The following 16 crop types were used for the analysis: barley, bean, cassava, cocoa, coffee, cotton, groundnut, maize, millet, potato, rice, sorghum, soybean, sugar beet, sugarcane, and wheat (cocoa is only included in M3‐Crop). In addition, seven groups of crops were used: banana & plantain, other fibers, other fruits, other pulses, other oil crops, sweet potato & yam, vegetables & melons (other fruits and vegetables & melons are only included in M3‐Crop, Table S1). In MapSPAM 2000, the groups fibers, oil crops, and pulses are defined as fibers: flax fiber & tow, hemp fiber & tow, kapok fiber, jute, jute‐like fibers, ramie, sisal, agave fibers nes (not elsewhere specified), abaca manila hemp, fiber crops nes, oil crops: coconut, oil palm fruit, olives, karite nuts (sheanuts), castor beans, sunflower, rapeseed, tung nuts, safflower seed, sesame, mustard seed, poppy seed, oilseeds nes (not elsewhere specified) and pulses: dry broad beans, dry peas, chickpea, cowpeas, pigeon peas, lentils, bambara beans, vetches, lupins, and pulses nes (not elsewhere specified). In M3‐Crop, the group fibers also contain coir and kapok seed, the group oil crops also contain hempseed, linseed, and melon seed.

We use data on livestock productivity (kg/km^2^) for two livestock products (meat, milk) and three types of animals (bovine, sheep and goats) as reported in Herrero et al. (2013). The data are for the year 2000, were converted to livestock production (kg), and then aggregated to a 30 arc minute resolution.

The harvested area fractions reported in the M3‐Crop data set were transformed to total area in hectares by multiplying with grid cell area. The livestock productivity data were converted to livestock production in kilogram. We excluded grid cells with more than 10% area equipped for irrigation (Siebert, Henrich, Frenken, & Burke, 2013) from the analysis to focus on rainfed agriculture solely. Areas of land cover classes, crop areas, and livestock production of all African land cells including Madagascar were summed up per rainfall class.

### 2.4 Measures of food security and farming diversity for household‐level analyses

We calculate food availability for 28,361 households across Africa (Table 1) by dividing the food energy potentially available by the energy requirements of a household following the approach of Frelat et al. (2016). Available energy is calculated from on‐farm produce and food purchases using off‐farm income and sales of farm products. A food availability value higher than one means that the farm household can generate enough energy with their activities to feed the family while a value of less than one means that the farm household is likely to be food insecure. Although a simple indicator of food security, it has been shown to be well related to other indicators of food security status and diet diversity across systems in Africa (Hammond et al., 2017). We also calculate an alternative measure of food security—food self‐sufficiency for which we exclude food bought from off‐farm income and sold farm produce. In addition to the six household surveys originally used in Frelat et al. (2016), we added 10,195 households from the World Bank Living Standards Measurement Study—Integrated Surveys on Agriculture (LSMS ISA), country programs for Ethiopia, Tanzania, Niger, and Uganda (World Bank, 2014). Four households were removed as outliers with food self‐sufficiency ratios exceeding the standard deviation more than 10 times.

**Table 1:** Household surveys used in this study

Dataset	Countries (ISO code)	No. of Households	No. of Geo‐referenced sites	Year(s)	Reference
FR16	BDI, BFA, COD, ETH, GHA, KEN, MLI, MWI, MOZ, NER, NGA, RWA, SEN, TZA, UGA, ZMB, ZWE	18,166	94	2006–2012	Frelat et al. (2016)
LSMS‐ISA	NER	2,272	214	2014	Niger National Institute of Statistics (2014)
	TZA	2,567	26	2010/2011	Tanzania National Bureau of Statistics (2011)
	ETH	2,654	296	2015/2016	Central Statistical Agency of Ethiopia (2015)
LSMS‐ISA	UGA	2,702	123	2010/2011	Uganda Bureau of Statistics (2010), Wichern, Van Wijk, and Descheemaeker (2017)

Farming diversity is the number of crops grown and the number of overall farming activities including livestock husbandry in a given year, irrespective of the economic importance of each activity. We calculate the overall farming diversity per household and the farming diversity per ha cropland and later discuss differences in these two measures. The individual crop and livestock types distinguished in each survey are different, reflecting the farming systems studied, but broad categories, for example, large and small ruminants, nonruminants, cash crops, and food crops, are covered equally well. Crop area information in the surveys considers that a household might own more than one plot or field but would usually only refer to the primary crop or land use. Hence, farming diversity needs to be understood as diversity across different plots or fields within 1 year not necessarily within. Further, farming diversity as defined here is different to agrobiodiversity in that we do not consider species that support food production indirectly, for example, soil organisms beneficial for soil fertility or insects, bacteria and fungi that control insect pests and diseases of plants and animals (Thrupp, 2000).

## 3 RESULTS

### 3.1 Are more diverse farming systems more food secure?

**Figure 1:**
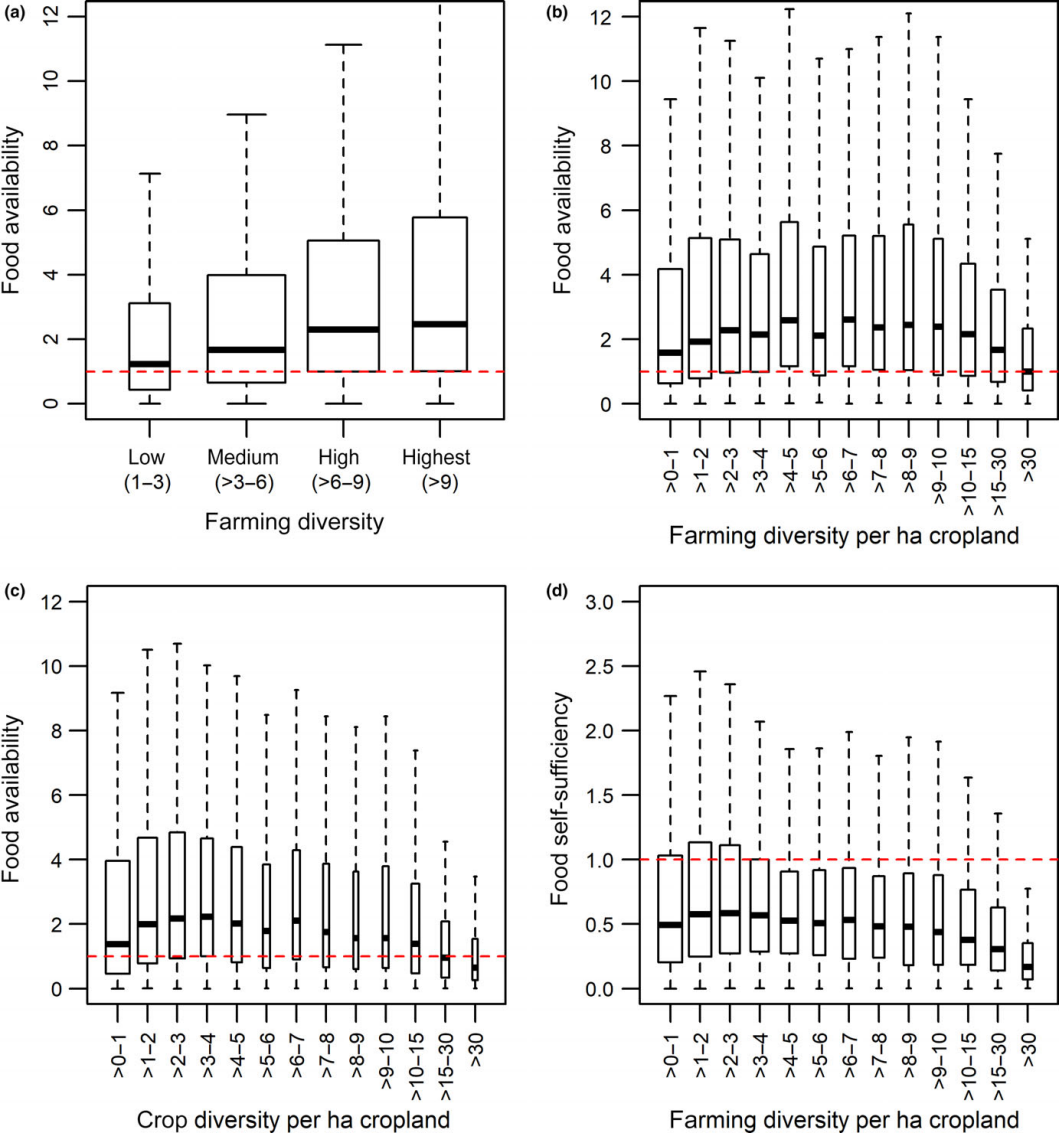
Farming diversity influences food security. Farming diversity is calculated by counting the number of crops grown and the number of livestock products shown as total count (a) and divided by cropland (b, d). Food availability as one dimension of food security is calculated as a ratio of energy available (sum of on‐farm consumption of food crops, food purchased using money earned through on‐farm, off‐farm activities) and energy requirements of a household. While (a) shows the basic relationship between diversity and food availability, the other three plots show the relationship while also controlling for size of cropland (b), livestock ownership (c) and income from farm sales and off‐farm activities (d). Please note that farm sizes can be very small, below 1 ha, so a maximum crop diversity of >30 can also relate to 10 crops grown on 0.3 ha. Boxplot widths are drawn proportional to the square roots of the number of households in each group. The red dashed line distinguishes households that meet their energy requirements (>1) from those that don't (<1). Outliers beyond the extremes of the whiskers (median ± 1.5 × IQR) are not shown. Please see the boxplot statistics in Table S3

We find that food availability on the household scale increases with farming diversity, irrespective of land size, livestock ownership, and off‐farm income, but only up to a certain level of diversity. The median food availability in the four farming diversity classes shown in Figure 1a increases, and there is strong evidence that the medians differ. This observation is based on the nonoverlapping 95% confidence interval around the medians of the four classes with different farming diversity approximated as median ± 1.57 × IQR/n^0.5^. The median and 95% confidence intervals for farming diversity in Figure 1a are low diversity (1.22, 1.11–1.32), medium diversity (1.59, 1.53–1.66), high diversity (2.19, 2.11–2.28), and highest diversity (2.42, 2.29–2.53). Also the Kruskal–Wallis rank sum test indicates that the differences between some of the medians are statistically significant.

Seventy‐five percentage of the households with high or highest farming diversity (>6 species) are able to meet their consumption needs, in contrast to only 55% of the households with low diversity (1–3 species; Figure 1a). The food secure households with high or highest farming diversity own on average two livestock units (e.g., two cows and three goats or sheep) and grow six different crops in contrast to the food secure households with low diversity that own only 0.1 livestock units (e.g., one goat or sheep) and grow only two different crops. This means that households with higher farming diversity tend to be more successful in meeting their consumption needs than households with lower diversity.

One reason for this is that the more diverse households also tend to own more cropland (see Benin, Smale, Pender, Gebremedhin, & Ehui, 2004; Makate, Wang, Makate, & Mango, 2016 and Figure S11), allowing them to grow a wider variety of crops which influences the overall farming diversity measure used here. Sixty percentage of all households studied own up to 2.2 ha, 80% own up to 5.5 ha. Adjusting for this effect, by calculating farming diversity per ha cropland, yields similar results with the exception of households with more than seven different crop and animal types per ha cropland (Figure 1b). Food availability scores differ between countries but with similar relationships between food availability and farming diversity (Figure S12).

Also using an alternative measure of food security—food self‐sufficiency for which we excluded food bought from off‐farm income and sold farm produce showed a similar relationship to farming diversity (Figure 1d). We control for off‐farm income and sold farm produce because it has been shown to increase household income which can influence food (e.g., Frelat et al., 2016; Sibhatu & Qaim, 2017) and farming diversity (McCord et al., 2015; Wencélius, Thomas, Barbillon, & Garine, 2016). Indeed, when excluding food bought from off‐farm income and sold farm produce, only 12%–27% of households are food secure. Households with a farming diversity of 2–3 per ha cropland have highest food self‐sufficiency scores but without the additional income from farm sales only 29% of households in this group are food secure (compared with 74% when off‐farm income and farm sales are included).

Also using an alternative measure of diversity, crop diversity for which we exclude livestock husbandry from the analysis shows an upward trend in food availability with increasing diversity and then a decline from 3 to 4 crops per hectare (Figure 1c). Thus, diversifying farming activities by growing more crops and engaging in a wider variety of farming activities can be a form of risk management or general livelihood strategy for a majority of households. In some situations, diversification using different crops may be more likely attributable to the benefits of rotating crops on the same area of land using the same amount of input (Barrett, Reardon, & Webb, 2001; Bationo & Ntare, 2000) than to risk management, given that yields are often correlated.

### 3.2 Diversification potential on the continental scale

**Figure 2:**
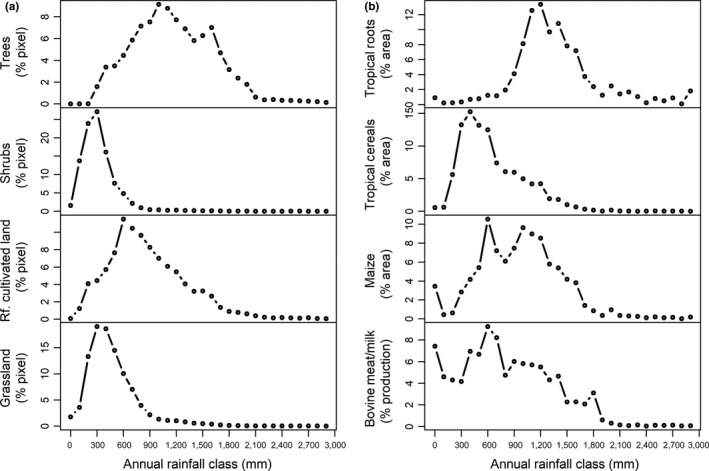
Rainfall constraints land cover and land use. Relationship between annual rainfall and MODIS land cover (a) and between annual rainfall and harvested area of rainfed tropical cereals, tropical roots and maize as in M3‐Crop and production of bovine meat and bovine milk (b). “Rf. cultivated land” is “Rainfed cultivated land.” The x‐axes show lower bounds of rainfall classes of 100 mm width. See supplementary materials for all crops and livestock products and for a comparison between M3‐Crop and MapSPAM2000 crop areas and between MODIS and GLC2000 land cover

In a next step, we explore the diversity of African farming systems on the continental scale using basic climatology. The spatial distribution of plants and animals globally is influenced by climate (Thomas, 2010; Woodward & Williams, 1987), and we are using these relationships here. Using two land cover products (Friedl et al., 2010; Fritz et al., 2003), we find that cultivated land is most likely to be located in areas with annual rainfall of 600–700 mm, which contrasts sharply with trees (>900 mm) and grassland and shrubs (300–400 mm; Figures 2a and S1; Table S2). The land cover class “cultivated land” comprises many different land uses, including a large number of individual crops that are grown and a variety of animals that are kept for meat, milk, draught, and insurance. Thus, we investigated the number of crops and livestock groups present over a gradient of annual rainfall using two different data sets reporting harvested areas of 23 crops (Monfreda et al., 2008; You et al., 2013) and meat and milk production from two animal groups (Herrero et al., 2013). Different crops and livestock are most likely to be present under different rainfall conditions, and these can be identified by the distribution of their area and production across rainfall gradients (Figures 2b, S2–S9). More specifically, we are interested in the peaks of the distributions in Figure 2b, as they represent the rainfall zone in which agricultural activity is highest. We then use the peaks to identify areas of overall high and low diversity. Many crops are most frequently grown at annual rainfall between 500 mm and 1,000 mm, while the peaks are lower for crops like wheat, pulses, forage, and sorghum/millet and higher for rice (Table S4, Figures S2 and S4). When testing the alternative data set reporting crop area, the peaks are identical or differ by only 100 mm in both data sets for all crops and crop groups except for soybean, potato, sugar beet and fiber crops. Livestock production generally peaks at annual rainfall of 600–700 mm (Figure 2b).

Several key observations can be made from the distributions in Figure 2. The constraints to agriculture at low rainfall are clear enough, but at high rainfall, few field crops tolerate prolonged water‐logging, which affects nutrient and water uptake, and land cover shifts increasingly to forest (Figure 2a). In addition, conditions of high rainfall and uniform warm temperatures are highly conducive to the development of many crop diseases (Anderson et al., 2004; Pautasso, Döring, Garbelotto, Pellis, & Jeger, 2012), which is part of the reason why crop losses due to disease in tropical humid regions may be double those in temperate regions (Ploetz, 1963). For several crops, more than one peak exists in at least one of the two crop area data sets (Table S4), probably as a result of genetically highly plastic crop types and/or grouping crops together with different moisture requirements or input levels.

**Figure 3:**
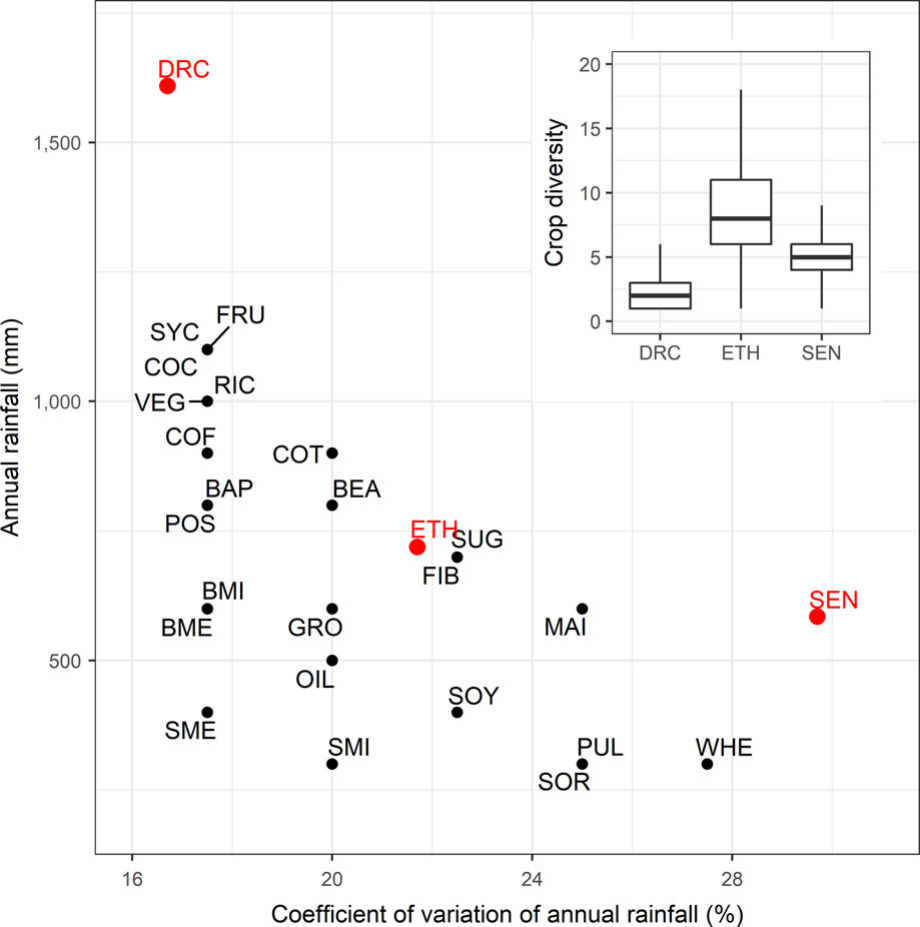
Rainfall zones with highest agricultural activity. Codes are BAP, Banana/Plantain; BEA, Beans; BME, Bovine meat; BMI, Bovine milk; COC, Cocoa; COF, Coffee; COT, Cotton; FIB, Fibers; FOR, Forage; FRU, Fruits; GRO, Groundnut; MAI, Maize; OIL, Other Oil Crops; POS, Potato/Sugarbeet; PUL, Other Pulses; RIC, Rice; SME, Sheep & goat meat; SMI, Sheep & goat milk; SOR, Sorghum/Millet; SOY, Soybean; SUG, Sugarcane; SYC, Sweetpotato/Yam/Cassava; VEG, Vegetables/Melons; WHE, Wheat/Barley; DRC, D.R. Congo, Walungu territory; ETH, Ethiopia, Southern Nations, Nationalities, and Peoples’ region; SEN, Senegal, Kaffrine region

A second constraint to agriculture is rainfall variability. Here, we define rainfall variability as the coefficient of variation (CV in %), which is statistically related to annual rainfall (Conrad, 1941). Rainfall variability decreases with increasing annual rainfall up to 1,500 mm after which this relationship disappears. Conrad (1941) first described this hyperbolic curve (Figure S10). Rainfall variability is a measure of the likelihood of extreme rainfall (drought and glut) and thus is closely related with crop failure. The majority of crops are most likely to be grown in areas with a rainfall CV of between 17% and 22% (Table S4, Figure 3). Some crops are outside this interval, for example, oil crops are most likely to be grown in areas with a rainfall CV of 15%; wheat is most likely to be grown at a CV of 27%. Livestock production peaks occur at similar CV as for the crop area.

These analyses indicate that the vast majority of rainfed agricultural activity takes places in rainfall zones between 500 and 1,000 mm with a mean of 747 mm and with rainfall variability between 17% and 22%. This is the high diversity window with 43% of cropland and 25% of pastures located in this interval.

For example, households in the Southern Nations, Nationalities, and Peoples’ region in Ethiopia with annual rainfall of 720 mm and rainfall CV of 22% grow on average eight crops a year, more than households in the Kaffrine region in Senegal and the Walungu territory in D.R. Congo that grow on average two and five crop species, respectively, and are located in rainfall zones with lower or higher rainfall variability (Figure 3). Crop diversity has a significant positive effect on food self‐sufficiency (Figure 1c), a key relationship underpinning the overall food availability, which is reflected in the farming diversity relation shown in Figure 1a. Therefore, farms with more diverse cropping systems are, in general, better able to feed themselves from their own produce.

**Figure 4:**
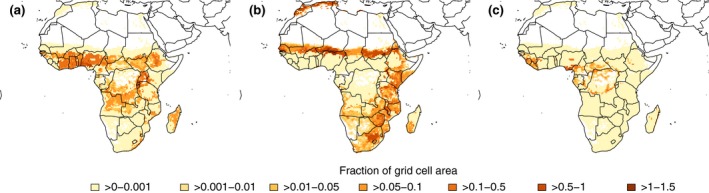
Agricultural areas with high and low farming diversity. Moderate to high farming diversity is found in areas with rainfall variability between 17% and 22% (a) whereas rainfall variability above 22% (b) or below 17% (c) limits farming diversity. Note that only cropland from the 23 crops analyzed here is shown, accounting for 155 Mha (77% of total arable land in Africa)

Spatially, farming diversity differs. Farming diversity is high in major agricultural areas of humid West Africa, and Ethiopia, Rwanda, Burundi and Uganda in East Africa (Figure 4a). We think of these areas as the ones with high diversification potentials, in that farmers have more choices what to grow and can more easily diversify their farming activities and, thus, are at lower risk to suffer negative impacts of future climate change. The potential for diversification by switching to a different crop or between crop and livestock farming in such areas with a CV between 17% and 22% is higher than in areas with lower or higher rainfall variability. An exception to the high farming diversity in West Africa is the cropping region along the Atlantic coast with high rainfall above 1,500 mm (Figure 4c), but which are unlikely to experience a shortage of rainfall in the future.

In contrast, cropping areas in East Africa south of Ethiopia, South Africa, the Sahel, and the Mediterranean coast of Morocco and Algeria are characterized by low farming diversity (Figure 4b). In these areas, already today rainfall limits agriculture and diversification potentials are low. Farmers in such area are less likely to diversify their farming activities by incorporating alternative crops or livestock. However, there are other means of adaptation such as adjusting to changing growing conditions by growing an earlier maturing crop cultivar. In semi‐arid environments with high rainfall variability, farmers might adjust by specializing to a few drought‐tolerant crop and livestock.

Future changes in rainfall and rainfall variability are difficult to project, but there is some evidence for a likely intensification of droughts in the 21st century in some seasons and areas in East and southern Africa (Funk et al., 2008), which can negatively impact cropping and livestock husbandry. In Eastern Africa, extreme precipitation changes such as droughts and heavy rainfall were experienced more frequently during the last 30–60 years (Williams & Funk, 2011), but the future direction of rainfall change is uncertain.

## 4 DISCUSSION

Our analyses have demonstrated that diversification will have an essential role to play in ensuring food security and stabilizing food production in Africa where possible. Our empirical analyses showed clear relations between farming diversity and food security, and a linkage to nutritional diversity also been demonstrated previously (e.g., Jones, Shrinivas, & Bezner‐Kerr, 2014), but there are mixed conclusions on how market orientation influences the relationship (Sibhatu, Krishna, & Qaim, 2015). This suggests the need of incentives to promote diversification, while intensifying production systems. Certification schemes, niche product markets, price and credit incentives could help promote the cultivation of nutrient‐rich, diverse foods in these environments.

At continental level, we can show where households are more or less likely to be able to adapt to changes in climate and climate variability (all other things being equal), because of their ability (or lack of ability) to make changes in crop and livestock types and to switch between them. While many other factors influence cropping and livestock production decisions, and there are other options for adaptation, we are able to demonstrate that rainfall and rainfall variability have explanatory power in relation to the current distribution of crop and livestock production in Africa. These simple, but robust relationships provide opportunities to rapidly assess feasible diversification options for different regions, thus offering a valuable input into policy and investment formulation. It was not possible to confirm the relationship between rainfall, rainfall variability, and farming diversity on the household scale. Some surveys assign GPS coordinates to larger sample units or clusters of households or only report the name of the respective district or province, partly to prevent identification of individual households and communities but also because GPS coordinate were not recorded during the time of the interview. This makes it difficult to combine household survey information with other spatial variables like rainfall or soil quality. However, there are methods for protecting confidential information and at the same time releasing useful spatially referenced household data (Perez‐Heydrich, Warren, Burget, & Emch, 2013).

While rainfall is a strong driver and rainfall variability is a good measure for identifying areas with high diversification potential, it is not the only factor determining crop choice and livestock production under current and future climate. Households might still be limited in their ability to diversify because of unfavorable soils, labor, input, and land constraints or because of their remote location without access to extension services that provide support for new crops or crop management techniques. It is estimated that about 64 Mha of African cropland (33% of total cropland) is of marginal quality (Cai, Zhang, & Wang, 2011) and 45 Mha of African land area is affected by nutrient depletion (1.5% of total land area and 23% of total cropland; Bai, Dent, Olsson, & Schaepman, 2008). This land, however, can often still be used for grazing animals and, therefore, contribute to farming diversity. Adjustments in farming practices will also require access to inputs and markets and an economic incentive for producing a certain crop or livestock product. Animal trypanosomiasis is prevalent in West and Central Africa and greatly affects livestock distributions (Meyer, Holt, Selby, & Guitian, 2016). Further multiple institutional, social, political, and economic barriers to adaptation in African agriculture will need to be removed. Crop and farming diversification need to be understood as part of overall livelihood diversification strategies (Mortimore & Adams, 2001; Newsham & Thomas, 2011), but this study clearly shows its importance for the current and future food security of smallholder farmers.

We found that diversification has a positive effect on food security, and although this is based on explorative results, the limits to diversification are at around 3–4 crops per ha cropland, or 4–7 crop and animal types per ha cropland. The limits to crop diversification are likely related to plants competing for light, water, and nutrients (Donald, 1963) in small fields with plant densities above optimum which affects growth and biomass production. When additional income from off‐farm employment and farm sales is not available, the most food secure households have only half of that farming diversity on average, highlighting again the importance of market access and employment options outside agriculture. However, it cannot be assumed that creating nonfarming employment opportunities will have uniform benefits or are uniformly desirable, for example, for the very poor or for enterprises operated by necessity (Block & Webb, 2001; Nagler & Naudé, 2017). Solutions for increasing food security need to consider both the agriculture and nonagricultural sectors, and we, here, focused on the relevance of farming diversity for achieving food security.

The needed adjustments in farming practices have significant policy and investment implications. Apart from requiring support from extension services, access to inputs and markets, and an economic incentive for producing a certain crop or livestock product, there is a need to shift the policy and research funding space so that it accommodates explicitly investments in diversification as well as improvements in the varieties of major staples that are more resilient to a changing climate. The Consortium of International Agricultural Research (CGIAR) allocated more than half of its crop‐specific resources to just two crops in 2012, rice and maize and more than 75% of its resources to five crops (Khoury & Jarvis, 2014). This shift will require acknowledgement that “different” can be an equally important solution as producing “more of the same.” Diversification will also have an essential role to play in ensuring nutritional security and tackling the problems of hidden hunger through micro‐nutrient deficiencies (Sibhatu et al., 2015) that will continue to affect increasing human population in Africa. Crop and farming diversification strategies need to be understood as a critical component of farmers’ adaptation to a changing climate.

## CONFLICT OF INTEREST

The authors have no conflict of interest to declare.

## AUTHOR CONTRIBUTION

MH and PT conceived the original idea of the study; KW, MvW, LS, and SF did the data analysis. MvW prepared indicators for calculating food security and diversity measures for LSMS Niger, Tanzania, and Ethiopia. JW prepared indicators for calculating food security and diversity measures for Uganda. LS and SF analyzed land cover and rainfall data and KW analyzed crop area, livestock production and rainfall data. KW, PT and MH prepared the first drafts, KW prepared the figures and the supplementary material and all authors were involved in discussing the results and reviewing the paper.

## References

[gcb14158-bib-0001] Altieri, M. A. (1999). The ecological role of biodiversity in agroecosystems. Agriculture, Ecosystems & Environment, 74(1–3), 19–31. Retrieved from http://www.sciencedirect.com/science/article/pii/S0167880999000286

[gcb14158-bib-0002] Anderson, P. K. , Cunningham, A. A. , Patel, N. G. , Morales, F. J. , Epstein, P. R. , & Daszak, P. (2004). Emerging infectious diseases of plants: Pathogen pollution, climate change and agrotechnology drivers. Trends in Ecology and Evolution, 19(10), 535–544. 10.1016/j.tree.2004.07.021 16701319

[gcb14158-bib-0003] Antwi‐Agyei, P. , Stringer, L. C. , & Dougill, A. J. (2014). Livelihood adaptations to climate variability: Insights from farming households in Ghana. Regional Environmental Change, 14(4), 1615–1626. 10.1007/s10113-014-0597-9

[gcb14158-bib-0004] Bai, Z. G. , Dent, D. L. , Olsson, L. , & Schaepman, M. E. (2008). Proxy global assessment of land degradation. Soil Use and Management, 24(3), 223–234. 10.1111/j.1475-2743.2008.00169.x

[gcb14158-bib-0005] Barrett, C. B. , Reardon, T. , & Webb, P. (2001). Nonfarm income diversification and household livelihood strategies in rural Africa: Concepts, dynamics, and policy implications. Food Policy, 26, 315–331. 10.1016/S0306-9192(01)00014-8

[gcb14158-bib-0006] Bationo, A. , & Ntare, B. R. (2000). Rotation and nitrogen fertilizer effects on pearl millet, cowpea and groundnut yield and soil chemical properties in a sandy soil in the semi‐arid tropics, West Africa. The Journal of Agricultural Science, 134(3), 277–284. 10.1017/S0021859699007650

[gcb14158-bib-0007] Below, T. B. , Mutabazi, K. D. , Kirschke, D. , Franke, C. , Sieber, S. , Siebert, R. , & Tscherning, K. (2012). Can farmers’ adaptation to climate change be explained by socio‐economic household‐level variables? Global Environmental Change, 22(1), 223–235. 10.1016/j.gloenvcha.2011.11.012

[gcb14158-bib-0008] Benin, S. , Smale, M. , Pender, J. , Gebremedhin, B. , & Ehui, S. (2004). The economic determinants of cereal crop diversity on farms in the Ethiopian highlands. Agricultural Economics, 31(2–3), 197–208. 10.1111/j.1574-0862.2004.tb00257.x

[gcb14158-bib-0009] Bezabih, M. , & Sarr, M. (2012). Risk preferences and environmental uncertainty: Implications for crop diversification decisions in Ethiopia. Environmental and Resource Economics, 53(4), 483–505. 10.1007/s10640-012-9573-3

[gcb14158-bib-0010] Bhatta, G. D. , Aggarwal, P. K. , Shrivastava, A. K. , & Sproule, L. (2016). Is rainfall gradient a factor of livelihood diversification? Empirical evidence from around climatic hotspots in Indo‐Gangetic Plains. Environment, Development and Sustainability, 18(6), 1657–1678. 10.1007/s10668-015-9710-6

[gcb14158-bib-0011] Block, S. , & Webb, P. (2001). The dynamics of livelihood diversification in post‐famine Ethiopia. Food Policy, 26, 333–350. 10.1016/S0306-9192(01)00015-X

[gcb14158-bib-0012] Bryan, E. , Ringler, C. , Okoba, B. , Roncoli, C. , Silvestri, S. , & Herrero, M. (2013). Adapting agriculture to climate change in Kenya: Household strategies and determinants. Journal of Environmental Management, 114, 26–35. 10.1016/j.jenvman.2012.10.036 23201602

[gcb14158-bib-0013] Cai, X. , Zhang, X. , & Wang, D. (2011). Land availability for biofuel production. Environmental Science & Technology, 45(1), 334–339. 10.1021/es103338e 21142000

[gcb14158-bib-0014] Central Statistical Agency of Ethiopia (2015). Ethiopia Socioeconomic Survey,Wave 3 (ESS3) 2015–2016. Addis Ababa, Ethiopia: The World Bank Retrieved from http://microdata.worldbank.org/index.php/catalog/2783/

[gcb14158-bib-0015] Conrad, V. (1941). The variability of precipitation. Monthly Weather Review, 69(1), 5–11. 10.1175/1520-0493(1941)069%3c0005:TVOP%3e2.0.CO;2

[gcb14158-bib-0016] Donald, C. M. (1963). Competition among crop and pasture plants. Advances in Agronomy, 15, 1–118.

[gcb14158-bib-0017] Ebi, K. , Padgham, J. , Doumbia, M. , Kergna, A. , Smith, J. , Butt, T. , & McCarl, B. (2011). Smallholders adaptation to climate change in Mali. Climatic Change, 108(3), 423–436. 10.1007/s10584-011-0160-3

[gcb14158-bib-0018] Frelat, R. , Lopez‐Ridaura, S. , Giller, K. E. , Herrero, M. , Douxchamps, S. , Djurfeldt, A. A. , … van Wijk, M. T. (2016). Drivers of household food availability in sub‐Saharan Africa based on big data from small farms. Proceedings of the National Academy of Sciences of the United States of America, 113(2), 458–463. 10.1073/pnas.1518384112 26712016 PMC4720294

[gcb14158-bib-0019] Friedl, M. , Sulla‐Menashe, D. , Tan, B. , Schneider, A. , Ramankutty, N. , Sibley, A. , & Huang, X. (2010). MODIS Collection 5 global land cover: Algorithm refinements and characterization of new datasets. Remote Sensing of Environment, 114(1), 168–182. 10.1016/j.rse.2009.08.016

[gcb14158-bib-0020] Fritz, S. , Bartholomé, E. , Belward, A. , Hartley, A. , Eva, H. , Mayaux, P. , … Defourny, P. (2003). Harmonisation, mosaicing and production of the Global Land Cover 2000 database (Beta Version). Luxembourg: Office for Official Publications of the European Communities.

[gcb14158-bib-0021] Funk, C. , Dettinger, M. D. , Michaelsen, J. C. , Verdin, J. P. , Brown, M. E. , Barlow, M. , & Hoell, A. (2008). Warming of the Indian Ocean threatens eastern and southern African food security but could be mitigated by agricultural development. Proceedings of the National Academy of Sciences of the United States of America, 105(32), 11081–11086. 10.1073/pnas.0708196105 18685101 PMC2497460

[gcb14158-bib-0022] Hammond, J. , Fraval, S. , van Etten, J. , Suchini, J. G. , Mercado, L. , Pagella, T. , … van Wijk, M. T. (2017). The Rural Household Multi‐Indicator Survey (RHoMIS) for rapid characterisation of households to inform climate smart agriculture interventions: Description and applications in East Africa and Central America. Agricultural Systems, 151, 225–233. 10.1016/j.agsy.2016.05.003

[gcb14158-bib-0023] Hazell, P. , & Wood, S. (2008). Drivers of change in global agriculture. Philosophical Transactions of the Royal Society B: Biological Sciences, 363(1491), 495–515. 10.1098/rstb.2007.2166 PMC2610166 17656343

[gcb14158-bib-0024] Herrero, M. , Havlík, P. , Valin, H. , Notenbaert, A. , Rufino, M. C. , Thornton, P. K. , … Obersteiner, M. (2013). Biomass use, production, feed efficiencies, and greenhouse gas emissions from global livestock systems. Proceedings of the National Academy of Sciences of the United States of America, 110(52), 20888–20893. 10.1073/pnas.1308149110 24344273 PMC3876224

[gcb14158-bib-0025] Hijmans, R. J. , Cameron, S. E. , Parra, J. L. , Jones, P. G. , & Jarvis, A. (2005). Very high resolution interpolated climate surfaces for global land areas. International Journal of Climatology, 25(15), 1965–1978. 10.1002/joc.1276

[gcb14158-bib-0026] Jones, A. D. , Shrinivas, A. , & Bezner‐Kerr, R. (2014). Farm production diversity is associated with greater household dietary diversity in Malawi: Findings from nationally representative data. Journal of Food Policy, 46, 1–12. 10.1016/j.foodpol.2014.02.001

[gcb14158-bib-0027] Jones, P. G. , & Thornton, P. K. (1999). Fitting a third‐order Markov rainfall model to interpolated climate surfaces. Agricultural and Forest Meteorology, 97(3), 213–231. 10.1016/S0168-1923(99)00067-2

[gcb14158-bib-0028] Jones, P. G. , & Thornton, P. K. (2013). Generating downscaled weather data from a suite of climate models for agricultural modelling applications. Agricultural Systems, 114, 1–5. 10.1016/j.agsy.2012.08.002

[gcb14158-bib-0029] Kazianga, H. , & Udry, C. (2006). Consumption smoothing? Livestock, insurance and drought in rural Burkina Faso. Journal of Development Economics, 79(2), 413–446. 10.1016/j.jdeveco.2006.01.011

[gcb14158-bib-0030] Khoury, C. K. , & Jarvis, A. (2014). The changing composition of the global diet: Implications for CGIAR research. *CIAT Policy Brief No. 18. CIAT Policy Brief* (Vol. 18).

[gcb14158-bib-0031] Lin, B. B. (2011). Resilience in agriculture through crop diversification: Adaptive management for environmental. Change, 61(3), 183–193. 10.1525/bio.2011.61.3.4

[gcb14158-bib-0032] Makate, C. , Wang, R. , Makate, M. , & Mango, N. (2016). Crop diversification and livelihoods of smallholder farmers in Zimbabwe: Adaptive management for environmental change. SpringerPlus, 5(1135), 18 10.1186/s40064-016-2802-4 27478752 PMC4951382

[gcb14158-bib-0033] Mary, A. L. , & Majule, A. E. (2009). Impacts of climate change, variability and adaptation strategies on agriculture in semi arid areas of Tanzania: The case of Manyoni District in Singida Region, Tanzania. African Journal of Environmental Science and Technology, 3(8), 206–218. 10.5897/AJEST09.099

[gcb14158-bib-0034] McCord, P. F. , Cox, M. , Schmitt‐Harsh, M. , & Evans, T. (2015). Crop diversification as a smallholder livelihood strategy within semi‐arid agricultural systems near Mount Kenya. Land Use Policy, 42, 738–750. 10.1016/j.landusepol.2014.10.012

[gcb14158-bib-0035] Meyer, A. , Holt, H. R. , Selby, R. , & Guitian, J. (2016). Past and ongoing tsetse and animal Trypanosomiasis control operations in five African Countries: A systematic review. PLoS Neglected Tropical Diseases, 10(12), 1–29. 10.1371/journal.pntd.0005247 PMC5222520 28027299

[gcb14158-bib-0036] Michler, J. D. , & Josephson, A. L. (2017). To specialize or diversify: Agricultural diversity and poverty dynamics in Ethiopia. World Development, 89, 214–226. 10.1016/j.worlddev.2016.08.011

[gcb14158-bib-0037] Monfreda, C. , Ramankutty, N. , & Foley, J. A. (2008). Harvested area and yields of 175 crops (M3‐crops data). Retrieved from http://www.earthstat.org/data-download/

[gcb14158-bib-0038] Mortimore, M. J. , & Adams, W. M. (2001). Farmer adaptation, change and ‘crisis’ in the Sahel. Global Environmental Change, 11, 49–57. 10.1016/S0959-3780(00)00044-3

[gcb14158-bib-0039] Nagler, P. , & Naudé, W. (2017). Non‐farm entrepreneurship in rural sub‐Saharan Africa: New empirical evidence. Food Policy, 67, 175–191. 10.1016/j.foodpol.2016.09.019 28413254 PMC5384454

[gcb14158-bib-0040] Newsham, A. J. , & Thomas, D. S. G. (2011). Knowing, farming and climate change adaptation in North‐Central Namibia. Global Environmental Change, 21(2), 761–770. 10.1016/j.gloenvcha.2010.12.003

[gcb14158-bib-0041] Niger National Institute of Statistics (2014). National survey on household living conditions and agriculture (ECVMA) 2014. Niamey, Niger: The World Bank Retrieved from http://microdata.worldbank.org/index.php/catalog/2676

[gcb14158-bib-0042] Pautasso, M. , Döring, T. F. , Garbelotto, M. , Pellis, L. , & Jeger, M. J. (2012). Impacts of climate change on plant diseases‐opinions and trends. European Journal of Plant Pathology, 133(1), 295–313. 10.1007/s10658-012-9936-1

[gcb14158-bib-0043] Perez‐Heydrich, C. , Warren, J. L. , Burget, C. R. , & Emch, M. E. (2013). Guidelines on the use of DHS GPS data. Spatial analysis reports no. 8. Calverton, Maryland. Retrieved from http://www.dhsprogram.com/pubs/pdf/SAR8/SAR8.pdf

[gcb14158-bib-0044] Ploetz, R. C. (1963). Diseases of tropical perennial crops: Challenging problems in diverse. Environments, 91(6). 10.1094/PDIS-91-6-0644 30780472

[gcb14158-bib-0045] Rufino, M. C. , Thornton, P. K. , Ng'ang'a, S. K. , Mutie, I. , Jones, P. G. , van Wijk, M. T. , & Herrero, M. (2013). Transitions in agro‐pastoralist systems of East Africa: Impacts on food security and poverty. Agriculture, Ecosystems & Environment, 179, 215–230. 10.1016/j.agee.2013.08.019

[gcb14158-bib-0046] Sibhatu, K. T. , Krishna, V. V. , & Qaim, M. (2015). Production diversity and dietary diversity in smallholder farm households. Proceedings of the National Academy of Sciences of the United States of America, 112(34), 10657–10662. 10.1073/pnas.1510982112 26261342 PMC4553771

[gcb14158-bib-0047] Sibhatu, K. T. , & Qaim, M. (2017). Rural food security, subsistence agriculture, and seasonality. PLoS One, 12(10), 1–15. 10.1371/journal.pone.0186406 PMC5648179 29049329

[gcb14158-bib-0048] Siebert, S. , Henrich, V. , Frenken, K. , & Burke, J. (2013). Global map of irrigation areas version 5. Bonn, Germany: Rheinische Friedrich‐Wilhelms‐University; Rome, Italy: Food and Agriculture Organization of the United Nations. Retrieved from http://www.fao.org/nr/water/aquastat/irrigationmap/

[gcb14158-bib-0049] Smith, N. J. H. (1990). Strategies for sustainable agriculture in the tropics. Ecological Economics, 2, 311–323. 10.1016/0921-8009(90)90018-P

[gcb14158-bib-0050] Tanzania National Bureau of Statistics (2011). Tanzania National Panel Survey Report (NPS) – Wave 2, 2010–2011. Dar es Salaam, Tanzania: The World Bank Retrieved from http://microdata.worldbank.org/index.php/catalog/1050/

[gcb14158-bib-0051] Thomas, C. D. (2010). Climate, climate change and range boundaries. Diversity and Distributions, 16(3), 488–495. 10.1111/j.1472-4642.2010.00642.x

[gcb14158-bib-0052] Thrupp, L. A. (2000). Linking agricultural biodiversity and food security: The valuable role of sustainable agriculture. International Affairs, 76(2), 265–281. Retrieved from http://www.jstor.org/stable/2626366?seq=1#page_scan_tab_contents 18383639 10.1111/1468-2346.00133

[gcb14158-bib-0053] Uganda Bureau of Statistics (2010). Uganda National Panel Survey (UNPS) 2010–2011. Kampala, Uganda: The World Bank Retrieved from http://microdata.worldbank.org/index.php/catalog/2166

[gcb14158-bib-0054] Waha, K. , Müller, C. , Bondeau, A. , Dietrich, J. P. P. , Kurukulasuriya, P. , Heinke, J. , & Lotze‐Campen, H. (2013). Adaptation to climate change through the choice of cropping system and sowing date in sub‐Saharan Africa. Global Environmental Change, 23(1), 130–143. 10.1016/j.gloenvcha.2012.11.001

[gcb14158-bib-0055] Wencélius, J. , Thomas, M. , Barbillon, P. , & Garine, E. (2016). Interhousehold variability and its effects on seed circulation networks: A case study from northern Cameroon. Ecology and Society, 21(1), 44.

[gcb14158-bib-0056] Wichern, J. , Van Wijk, M. T. , & Descheemaeker, K. (2017). Food availability and livelihood strategies among rural households across Uganda. Food Security, 9, 1385–1403. 10.1007/s12571-017-0732-9

[gcb14158-bib-0057] Williams, A. P. , & Funk, C. (2011). A westward extension of the warm pool leads to a westward extension of the Walker circulation, drying eastern Africa. Climate Dynamics, 37(11–12), 2417–2435. 10.1007/s00382-010-0984-y

[gcb14158-bib-0058] Woodward, F. I. , & Williams, B. G. (1987). Climate and plant distribution at global and local scales. Theory and Models in Vegetation Science, 69(1/3), 189–197. 10.1007/978-94-009-4061-1

[gcb14158-bib-0059] World Bank (2014).Living standards measurement study. Retrieved from http://go.worldbank.org/UJ3E1I2850

[gcb14158-bib-0060] You, L. , Crespo, S. , Guo, Z. , Koo, J. , Ojo, W. , Sebastian, K. , … Wood‐Sichra, U. (2013). Spatial Production Allocation Model (SPAM) 2000 Version 3 Release 2. Retrieved from http://mapspam.info/data/

