# Supplementary material for: Agricultural diversification as an important strategy for achieving food security in Africa
Source: Glob Chang Biol. 2018 Apr 25;24(8):3390–400. doi: 10.1111/gcb.14158 (PMC6055696; doi:10.1111/gcb.14158)
Supplement: Supplementary file 1 [file GCB-24-3390-s001.pdf]

Supplementary Materials for

Agricultural diversification as an important strategy for achieving food security in Africa

Authors: Katharina Waha[1*], Mark T. van Wijk[2], Steffen Fritz[3], Linda See[3], Philip K. Thornton[1,4], Jannike Wichern[5], Mario Herrero[1]

Affiliations:
[1] CSIRO Agriculture & Food, 306 Carmody Rd, St Lucia, QLD, Australia

[2] International Livestock Research Institute (ILRI), Livestock Systems and the Environment, Nairobi 00100, Kenya

[3] IIASA, International Institute for Applied Systems Analysis (IIASA), Schlossplatz 1, A-2361 Laxenburg, Austria

[4] CGIAR Research Program on Climate Change, Agriculture and Food Security (CCAFS), ILRI, PO Box 30709, Nairobi 00100, Kenya

[5] Plant Production Systems, Wageningen University & Research, 6700, AK Wageningen, The Netherlands

Correspondence to: katharina.waha@csiro.au

This PDF file includes:

Figures S1-S12

Tables S1-S4

References

Table S1
Comparison of harvested crop areas (ha) in Africa as reported in MapSPAM 2000 and M3-Crop.

Crop/Crop Group	MapSPAM 2000	M3-Crop
Individual crops		
Barley	3,918,608	3,559,759
Bean	5,125,568	4,002,190
Cassava	10,202,024	9,663,990
Cocoa	-	4,549,768
Coffee	2,496,553	2,338,683
Cotton	4,217,975	3,803,926
Groundnut	8,188,165	8,206,634
Maize	26,193,814	22,644,964
Millet	17,957,129	17,389,348
Potato	1,230,140	923,248
Rice	6,810,709	6,739,728
Sorghum	21,059,810	19,504,675
Soybean	1,610,069	905,159
Sugarbeet	107,455	114,498
Sugarcane	1,194,620	1,149,725
Wheat	8,309,652	8,030,723
Crop groups		
Banana & Plantain	5,245,118	4,912,245
Other Fibers w/o cotton	357,755	367,693
Other Fruits w/o banana & plantain	-	3,600,785
Other Pulses w/o beans	11,031,701	12,427,821
Other Oil Crops w/o groundnut & soybean	12,490,151	10,677,198
Sweet Potato & Yam	4,722,205	5,554,715
Vegetables & Melons	-	4,416,681
**Total**	152,469,221	155,484,156

The difference is less than 10% between the two data sets for all crops and crop groups except maize, other pulses, other oil crops, sweet potato & yam, bean, potato and soybean. Total harvested crop areas in Africa for the selected crops are 152 Mha in MapSPAM2000 and 155 Mha in M3-Crop, which accounts for 75% and 77% respectively of the total arable land in Africa as reported in FAOStat in 2000 (202 Mha).

Table S2
Simplification of MODIS land cover classes.

MODIS Land Cover Classes	Simplified Classes
Evergreen needleleaf forest<br>Evergreen broadleaf forest<br>Deciduous needleleaf forest<br>Deciduous broadleaf forest<br>Mixed Forest<br>Woody Savannas	Tree Class

Savannas	
Closed shrublands<br>Open shrublands	Shrubs
Grasslands	Grasslands
Croplands<br>Cropland/Natural Vegetation Mosaic	Cultivated Land
Barren or Sparsely Vegetated<br>Water<br>Snow and Ice<br>Urban and Built-up<br>Barren or Sparsely Vegetated<br>Permanent Wetlands<br>Unclassified	Not regarded

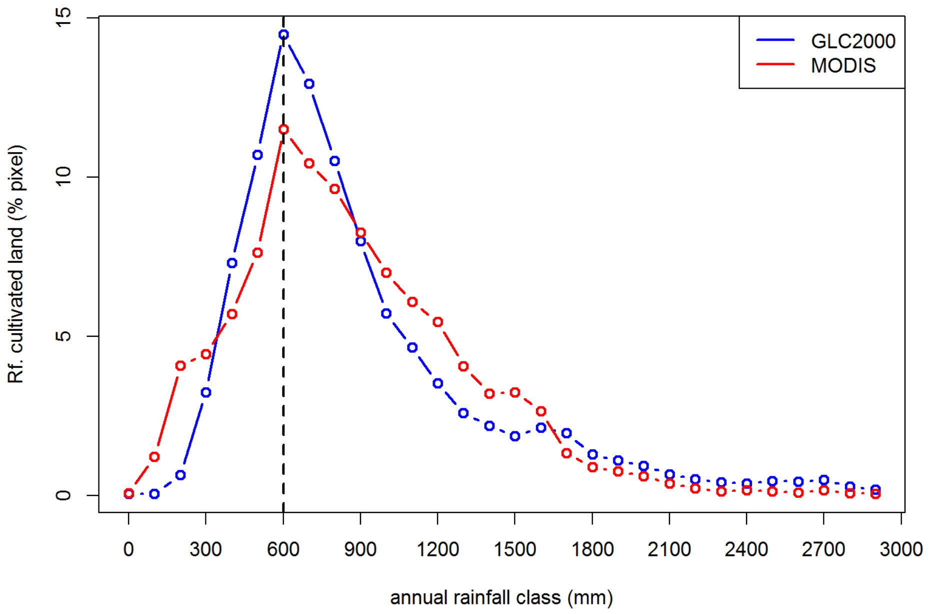

Fig. S1
Rainfed cultivated area (GLC2000 and MODIS) related with total annual rainfall. For the GLC2000 land cover product, cultivated land was determined as the aggregation of three classes land cover classes: Cultivated and managed areas, and two mosaic classes that include cropland. Both frequency distribution curves have a similar shape and peak at 600-700mm total annual rainfall.

Table S3
Statistics for boxplots show in Figure 1: lower and upper whisker (median +/- 1.5 x IQR), lower and upper 'hinge' (close to first and third quartile), median and number of household per class.

**Panel A - farming diversity vs. food availability**				
	1	2	3	4
l. whisker	0	0	0	0
l. hinge	0.4	0.7	1	1
median	1.2	1.7	2.3	2.5
u. hinge	3.1	4	5.1	5.8
u. whisker	7.1	9	11.1	12.9
N	1463	5274	4451	2577

% food secure	55.6	65.0	74.7	75.0

Classes 1 to 4 refer to farming diversity classes ‘low’ (1), ‘medium’ (2), ‘high’ (3) and ‘highest (4)

Panel B - farming diversity per ha cropland vs. food availability

	1	2	3	4	5	6	7	8	9	10	11	12	13
l. whisker	0	0	0	0	0	0	0	0	0	0	0	0	0
l. hinge	0.6	0.8	1	1	1.2	0.9	1.2	1.1	1	0.9	0.9	0.7	0.4
median	1.6	1.9	2.3	2.1	2.6	2.1	2.6	2.4	2.4	2.4	2.2	1.7	1
u. hinge	4.2	5.1	5.1	4.6	5.6	4.9	5.2	5.2	5.6	5.1	4.3	3.5	2.3
u. whisker	9.4	11.7	11.2	10.1	12.2	10.7	11	11.4	12.1	11.4	9.4	7.8	5.1
N	2310	2073	1597	1345	1195	582	626	519	390	359	884	801	392
% food secure	62.9	69.5	74.1	74.3	78.8	72.0	77.6	76.7	76.7	72.4	72.6	66.2	49.6

Classes 1 to 13 refer to farming diversity classes of >0-1, >1-2, >2-3, >3-4, >4-5, >5-6, >6-7, >7-8, >8-9, >9-10, >10-15, >15-30 and >30

Panel C - crop diversity per ha cropland vs. food availability

	1	2	3	4	5	6	7	8	9	10	11	12	13
l. whisker	0	0	0	0	0	0	0	0	0	0	0	0	0
l. hinge	0.5	0.8	0.9	1	0.8	0.6	0.9	0.7	0.6	0.6	0.5	0.3	0.3
median	1.4	2	2.2	2.2	2	1.8	2.1	1.7	1.6	1.6	1.4	1	0.7
u. hinge	4	4.7	4.8	4.7	4.4	3.8	4.3	3.9	3.6	3.8	3.2	2.1	1.5
u. whisker	9.2	10.5	10.7	10	9.7	8.5	9.3	8.4	8.1	8.4	7.4	4.6	3.5
N	5116	3371	2042	1370	1003	464	474	318	243	338	562	577	291
% food secure	58.5	69.2	72.6	75.3	69.7	66.2	72.2	65.7	63.0	64.5	57.3	48.2	38.0

Classes 1 to 13 refer to crop diversity classes of >0-1, >1-2, >2-3, >3-4, >4-5, >5-6, >6-7, >7-8, >8-9, >9-10, >10-15, >15-30 and >30

Panel D - farming diversity per ha cropland vs. food self-sufficiency

	1	2	3	4	5	6	7	8	9	10	11	12	13
l. whisker	0	0	0	0	0	0	0	0	0	0	0	0	0
l. hinge	0.2	0.2	0.3	0.3	0.3	0.3	0.2	0.2	0.2	0.2	0.2	0.1	0.1
median	0.5	0.6	0.6	0.6	0.5	0.5	0.5	0.5	0.5	0.4	0.4	0.3	0.2
u. hinge	1	1.1	1.1	1	0.9	0.9	0.9	0.9	0.9	0.9	0.8	0.6	0.4
u. whisker	2.3	2.5	2.4	2.1	1.9	1.9	2	1.8	1.9	1.9	1.6	1.4	0.8
N	2309	2073	1597	1345	1195	582	626	519	390	359	884	801	392
% food secure	25.7	29.6	29.1	25.1	21.7	21.8	21.9	19.7	21.3	19.2	15.3	11.1	3.1

Classes 1 to 13 refer to farming diversity classes of >0-1, >1-2, >2-3, >3-4, >4-5, >5-6, >6-7, >7-8, >8-9, >9-10, >10-15, >15-30 and >30

Table S4
Annual rainfall and CV of rainfall 'peaks' for crops and livestock products.

	**Annual rainfall (mm)**		**CV (%) of rainfall**	
Crop/Crop Group	1st peak	2nd peak	1st peak	2nd peak
Other pulses w/o bean	300-400	-	25-27.5	
Wheat/Barley	300-400	-	27.5-30	
Sorghum/Millet	300-400	-	25-27.5	20-22.5
Soybean	400-500	-	22.5-25	-
Other oil crops w/o groundnut & soybean	500-600	800-1000	20-22.5	25-27.5
Groundnut	600-700	-	20-22.5	25-27.5
Maize	600-700	900-1000	25-27.6	17.5-20
Other fibers w/o cotton	700-800	-	22.5-25	-
Sugarcane	700-900	-	22.5-25	-
Potato/Sugarbeet	800-900	-	17.5-20	
Banana & Plantain	800-900	1000-1100	17.5-20	-
Bean	800-900	1000-1100	20-22.5	-
Coffee	900-1000	1100-1200	17.5-20	-
Cotton	900-1000	-	20-22.5	25-27.5
Rice	1000-1100	1400-1500	17.5-20	
Vegetables & Melons	1000-1100	-	17.5-20	-
Other Fruits w/o banana & plantain	1100-1200	-	17.5-20	-
Cocoa	1100-1200	-	17.5-20	-
Sweetpotato & Yam/Cassava	1100-1200	-	17.5-20	-
Livestock				
Sheeps & goats milk	300-400		20-22.5	25-27.5
Sheeps & goats meat	400-500		17.5-20	25-27.5
Bovine milk	600-700		17.5-20	25-27.5
Bovine meat	600-700		17.5-20	25-27.5

Based on harvested crop area from MapSPAM 2000 except for Other fruits, Vegetables & Melons and Cocoa (data based on M3-Crop) and Herrero et al. 2013. Peaks in annual rainfall and rainfall variability do not differ between the two crop area data sets for most crops except for soybean, potato, sugar beet and other fibers. Where no clear single peak could be identified the second peak is reported as well.

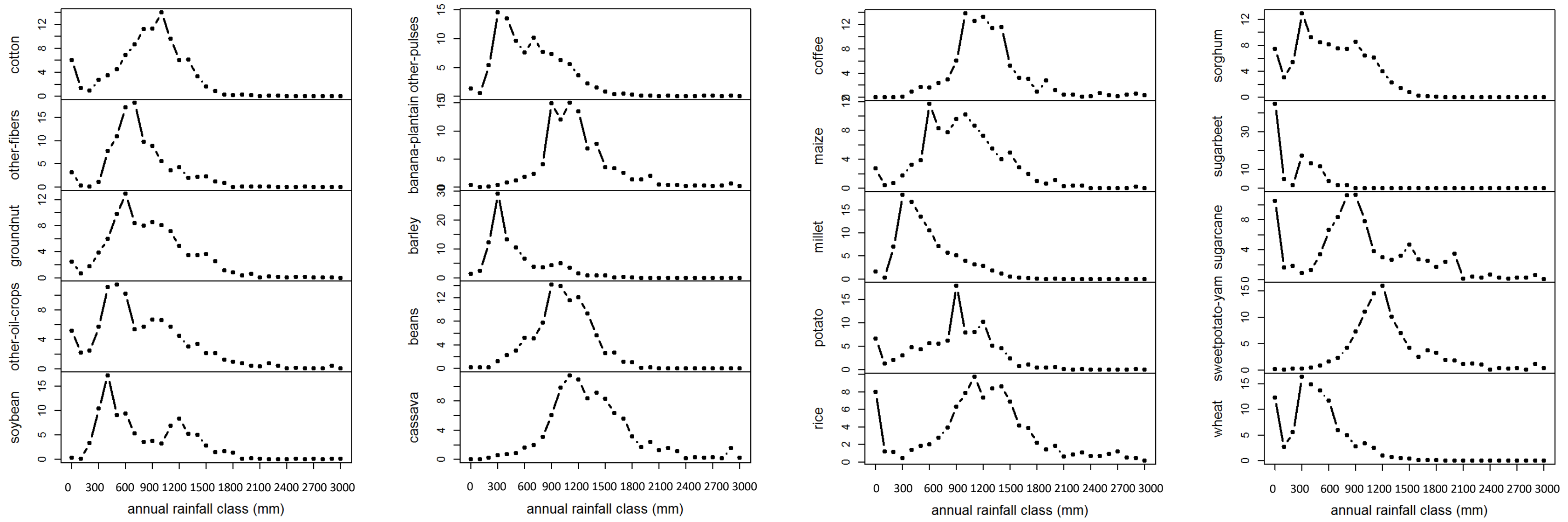

Fig. S2 Relationship between annual rainfall (mm) and crop area (%) as in MapSPAM2000

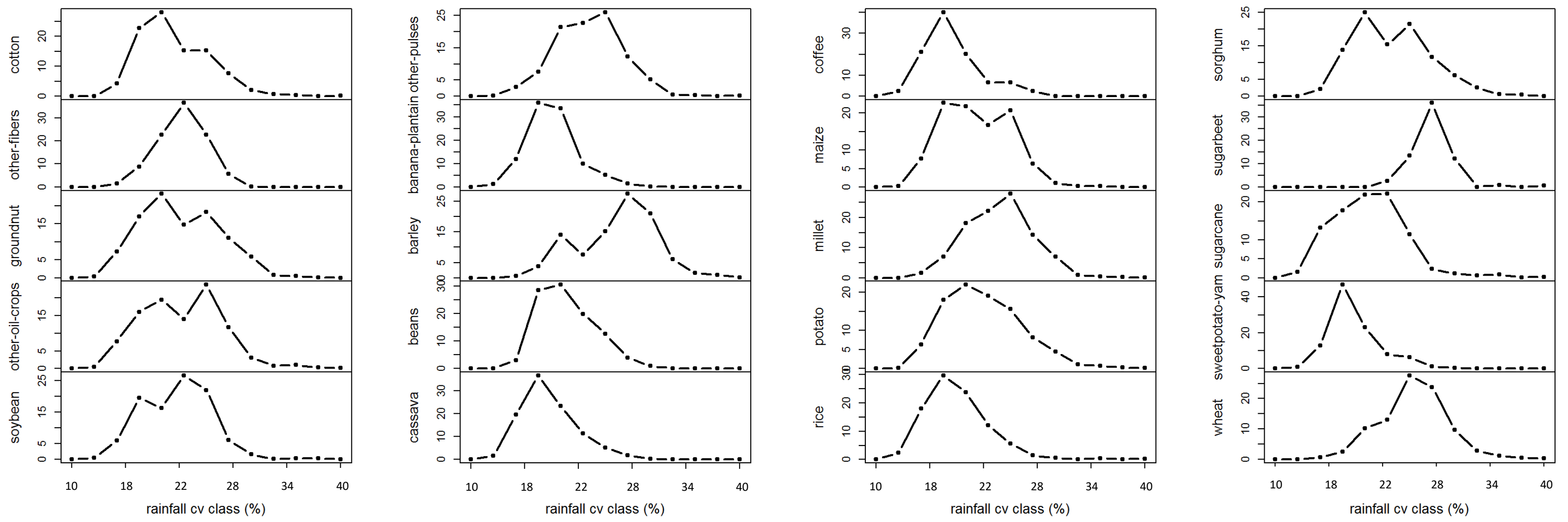

Fig. S3 Relationship between rainfall CV (%) and crop area (%) as in MapSPAM2000

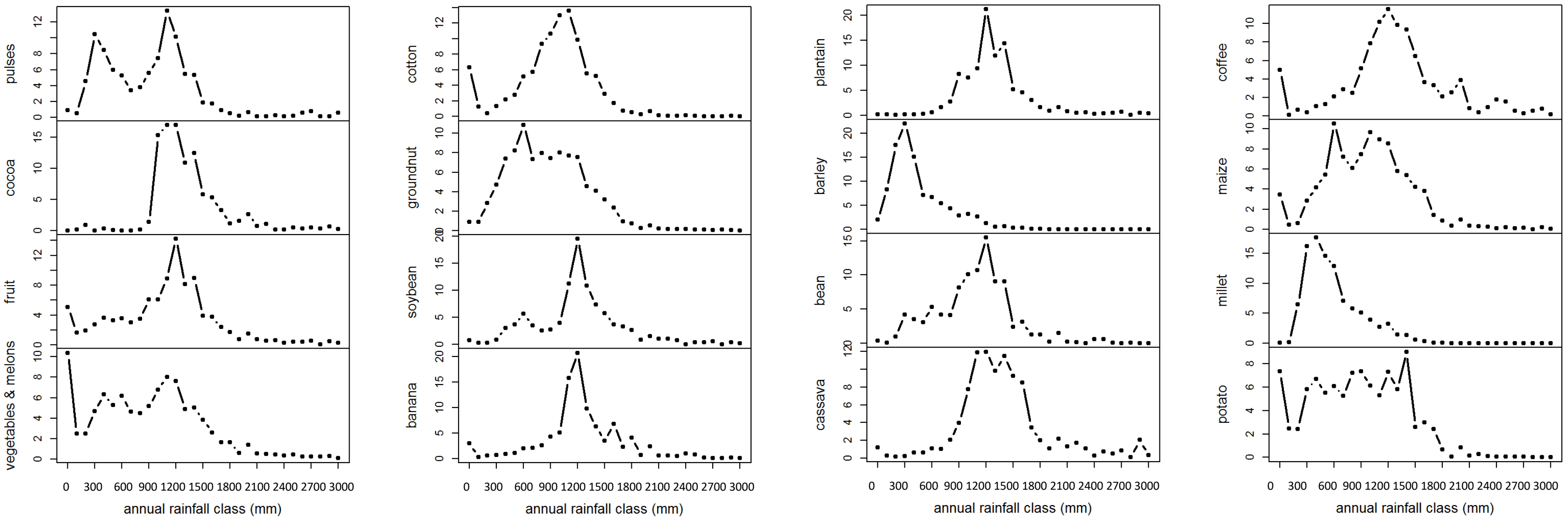

Fig. S4 Relationship between annual rainfall (mm) and crop area (%) as in M3-Crop

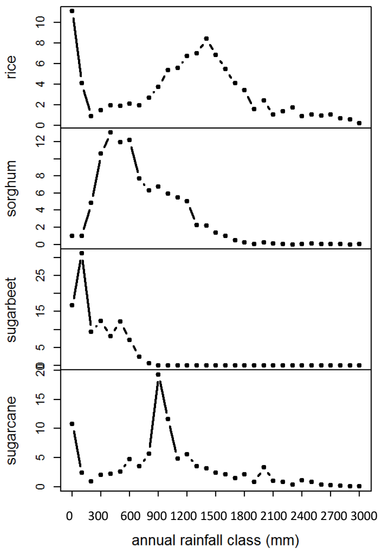

Fig. S5 Relationship between annual rainfall (mm) and crop area (%) as in M3-Crop (Continuation)

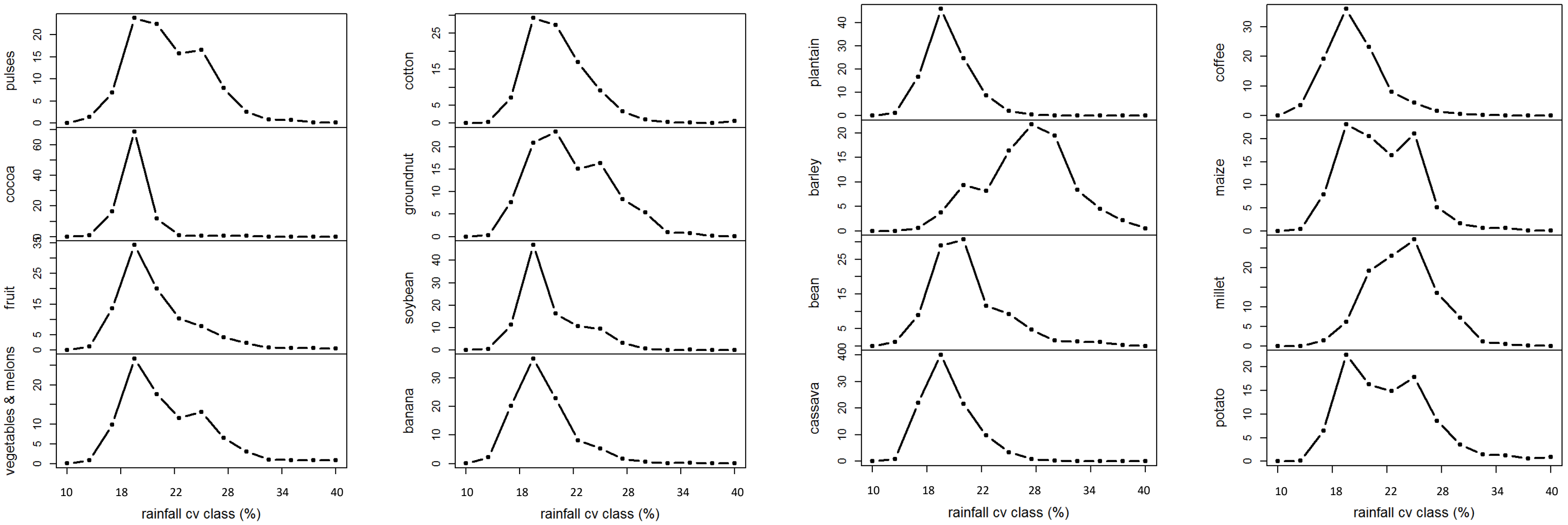

Fig. S6 Relationship between rainfall CV (%) and crop area (%) as in M3-Crop

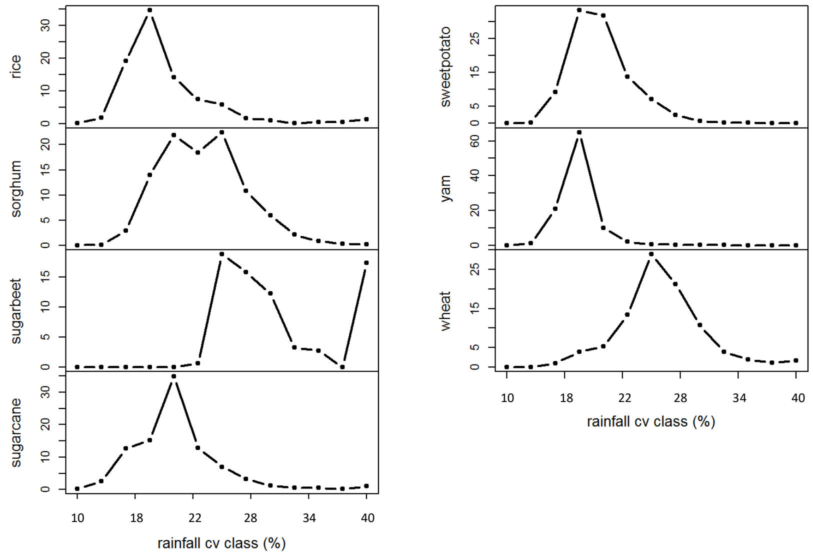

Fig. S7 Relationship between rainfall CV (%) and crop area (%) as in M3-Crop (Continuation)

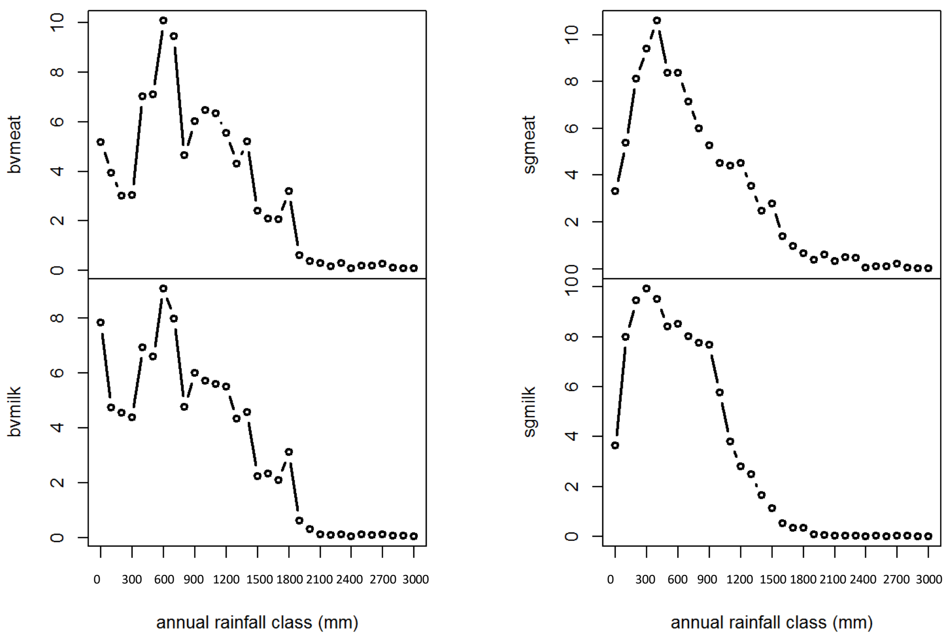

Fig. S8
Relationship between annual rainfall (mm) and livestock production (%): bv for bovine, sg for sheep & goat

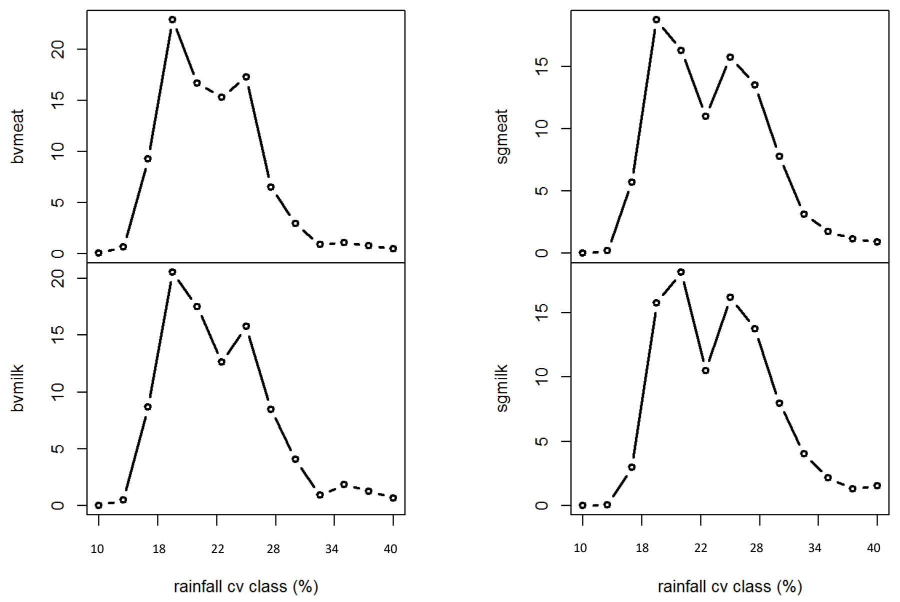

Fig. S9
Relationship between rainfall CV (%) and livestock production (%): bv for bovine, sg for sheep & goat

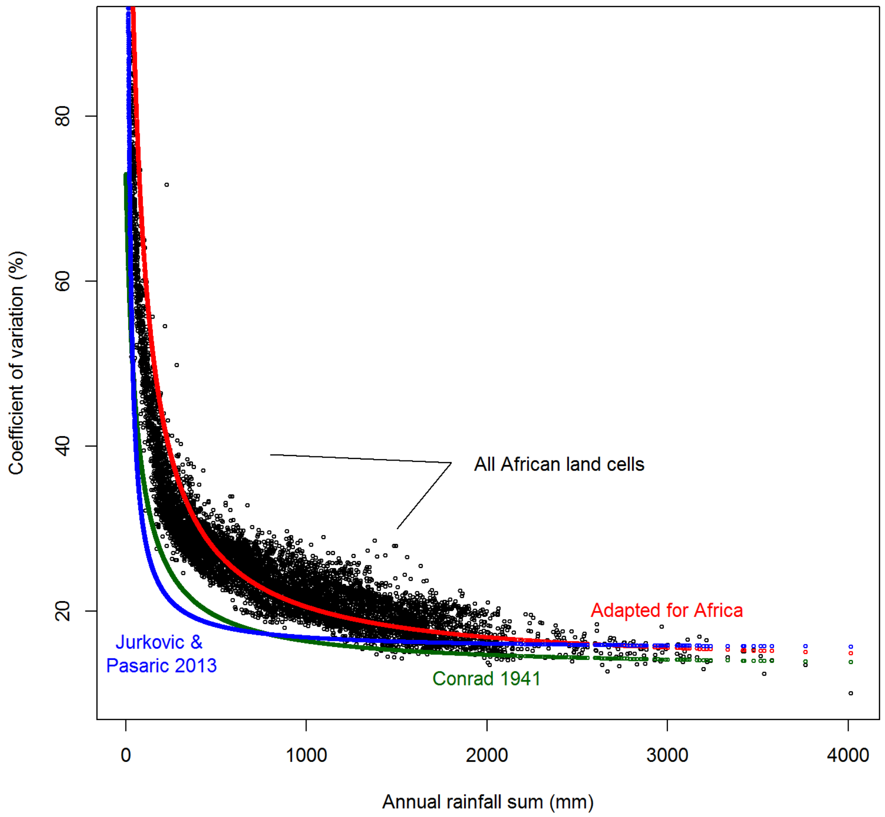

Fig. S10
Mathematical relation between rainfall variability and annual rainfall. Rainfall variability is modelled as: 3600 / (Annual rainfall + 60) + 13 in Conrad (1941) using global rainfall data, 1495 / (Annual rainfall + 5.52) + 15.37 in Jurkovic & Pasaric (2013) using global rainfall data and 8000 / (Annual rainfall + 60) + 13 when adapted for rainfall data for African land cells used in this study. An annual rainfall sum of 700mm relates to a coefficient of variation of 23.5%.

There is a strong mathematical relation between annual rainfall and inter-annual rainfall variability (R2 = 0.81 in Jurkovic & Pasaric 2013). This relation was first described as a hyperbolic curve in Conrad (1941) where starting from 1500mm the variability of the annual rainfall was found to be independent of the annual sum. The hyperbolas shown in Fig. S3 relate to global data from 384 weather stations in Conrad (1941) and several thousand weather stations in Jurkovic & Pasaric (2013) and therefore do not fit the African data. When adapted for African land cells rainfall variability can be modelled as 8000 / (Annual rainfall + 60) + 13. An annual rainfall sum of 700mm relates to a coefficient of variation of 23.5%.

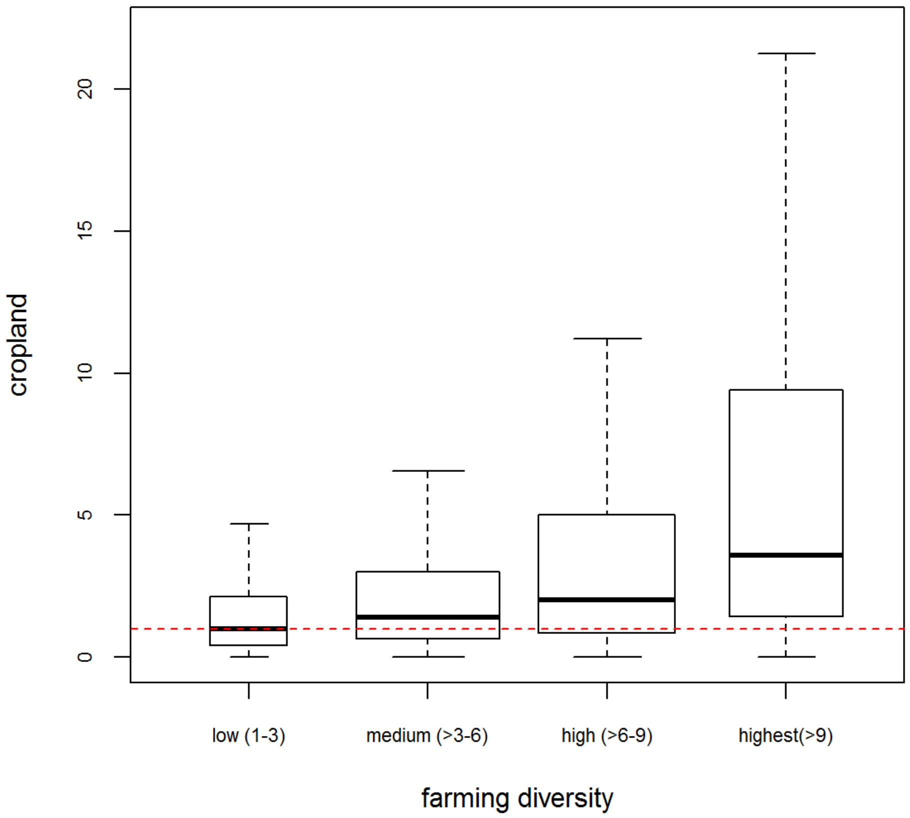

Fig. S11
Cropland and farming diversity relation. Farming diversity is calculated by counting the number of crops grown and the number of livestock products. Boxplot widths are drawn proportional to the square-roots of the number of households in each group. The red dashed line distinguishes households that meet their energy requirements (>1) from those that don't (<1).

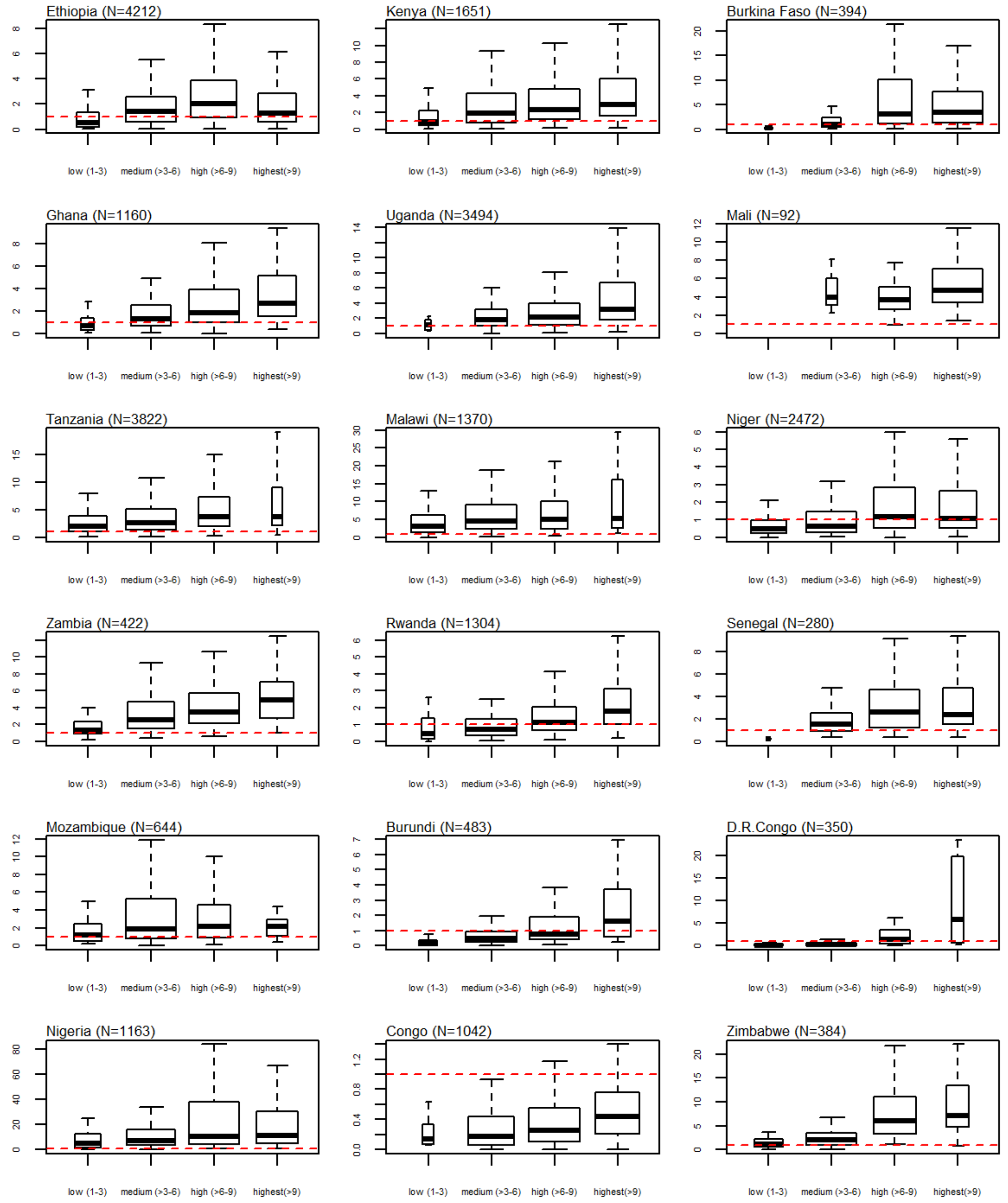

Fig. S 12

The relationship between farming diversity and food availability per country. Farming diversity is calculated by counting the number of crops grown and the number of livestock products. Numbers in brackets are the number of households surveyed in each country.

References

Conrad V (1941) The variability of precipitation. *Monthly Weather Review*, **69**, 5–11.

Sokol Jurković R, Pasarić Z (2013) Spatial variability of annual precipitation using globally gridded data sets from 1951 to 2000. *International Journal of Climatology*, **33**, 690–698.
